# Letter from Dr. Stovall, of Terrell

**Published:** 1888-12

**Authors:** A. J. Stovall


### LETTER FROM DR. STOVALL.

[We publish the following at Dr. Stovall's request. We did not know the gentleman at the time, and as we published only the action of the convention, we could have had no personal feeling in the matter, and no desire to do him injury.—ED.]

*Pvblisher Daniel's Texas Medical Journal, Austin, Texas:*

DEAR SIR—I was surprised to see in your JOURNAL, publishing the proceedings of the Texas State Medical Association of 1887, my name published as a quack and empiric, in connection with W. G. Hardin, in the form of a report of a committee, signed by Drs. H. L. Parsons and W. W. Reeves.

I had given Dr. Parsons credit for having too much sense to have had that charge published against me, knowing as he does that I hold the following certificate, signed by himself:

TERRELL, TEXAS, February 4, 1881.

"Having this day examined Dr. A. J. Stovall as to his proficiency in anatomy, materia medica, chemistry and general medicine, and finding him possessed of the necessary qualifications to entitle him to practice medicine, I hereby authorize him to do so."

(Signed) H. L. PARSONS, M. D.,
Member Ex. Board, 8th Jud. Dist., Texas.

Since he signed the above, I have graduated from the University of Louisville, Medical Department, 1882, also received diploma on special courses.

The Medical Association can publish what they please against their own members, so far as I am concerned, but as I am not a member of the Association, they have no right to publish me as a quack.

I have lived in Kaufman county for twenty-one years, and have enjoyed a lucrative practice since I graduated; and I think my character as a man and a physician will bear as close a scrutiny as any physician in the State, as will my success in the practice of medicine.

I am one of the firm composing the "Stovall Medicine Company," which advertises, which, I suppose, was the grounds for the charge of quackery. We do not sell or manufacture any patent medicines. Our medicines are all made from well-known formulas. Known and used by all the profession, there are no secrets about any of it. If this constitutes a quack, then the Association had better commence to weed the quacks out of their own organization. I know that some of the most eminent physicians in the State are members of the Association, and I do not wish to cast any reflections on their good name; and I don't think they would knowingly inflict an injury on anyone. Dr. Parsons is liable to door say anything, and a journal publishing anything emanating from his pen is liable to get into trouble.

A. J. STOVALL, M. D.

[Dr. Stovall is a member in good standing in Terrell Medical Society.—ED.]

